# High Prevalence, Genetic Diversity and Temporal Differentiation of *Plasmodium vivax* in a Remote Hard-to-Reach Community from the Peruvian Amazon Region

**DOI:** 10.4269/ajtmh.24-0662

**Published:** 2025-08-19

**Authors:** Roberson Ramirez, Katherine Torres, Pamela Rodríguez, Carlos Acosta, Mitchel Guzmán-Guzmán, Alejandro Llanos-Cuentas, Joseph M. Vinetz, Ananias A. Escalante, Dionicia Gamboa

**Affiliations:** ^1^ Amazonian International Center of Excellence for Malaria Research, Laboratorios de Investigación y Desarrollo, Facultad de Ciencias e Ingeniería, Universidad Peruana Cayetano Heredia, Lima, Peru;; ^2^ Instituto de Medicina Tropical “Alexander von Humboldt”, Universidad Peruana Cayetano Heredia, Lima, Peru;; ^3^ Departamento de Ciencias Celulares y Moleculares, Facultad de Ciencias e Ingeniería, Universidad Peruana Cayetano Heredia, Lima, Peru;; ^4^ Section of Infectious Diseases, Department of Internal Medicine, Yale School of Medicine, New Haven, Connecticut;; ^5^ Institute for Genomics and Evolutionary Medicine, Temple University, Philadelphia, Pennsylvania

## Abstract

Assessing parasite population genetic diversity and structure in remote areas is essential for understanding malaria transmission and guiding interventions toward elimination. This study monitored the genetic diversity and population structure of *Plasmodium vivax* as part of a longitudinal surveillance in Santa Emilia, a hard-to-reach community in Loreto, Peru. A total of 221 of 3,434 *P. vivax* samples collected through active and passive case detection between 2015 and 2016 were genotyped using 16 neutral microsatellites. Additionally, 139 genotyped samples from 2013, previously reported, were included for comparison. Malaria prevalence (microscopic and submicroscopic), genetic diversity, population differentiation, structure, bottleneck analysis, and relatedness between years were evaluated. We found 56% *P. vivax* prevalence by quantitative real-time polymerase chain reaction, with 44% submicroscopic infections in 2015 and 2016. Genetic diversity and population differentiation were high between 2013, 2015, and 2016. Parasites from 2015 to 2016 had a lower Jost *D*. In 2013 and 2015, more than 40% of infections were polyclonal infections, but only 29% were polyclonal infections in 2016. Moderate linkage disequilibrium was found over time. Four populations were detected in 2013, 2015, and 2016, with increasing admixture in 2015–2016. Genetically related parasites with clonal expansion suggest that there was no recent bottleneck. Santa Emilia has a persistent high genetic diversity and structured, temporally differentiated clonal populations over the time periods of the study. This analysis highlights the complexity of parasite dynamics in this remote area of malaria transmission, making it a challenging area for the malaria elimination plan in Peru.

## INTRODUCTION

Malaria remains a public health problem in tropical and subtropical countries. According to the WHO, nearly 247 million total cases were reported in 2021, and 0.6 million cases were in the Americas.^1^ In Peru, malaria cases in the Amazonian department of Loreto increased by approximately 54% between 2011 and 2016,^2^ despite the success achieved by Control de la Malaria en las Zonas Fronterizas de la Región Andina: Un Enfoque Comunitario – PAMAFRO (2005–2010), a program sponsored by the Global Fund.^3^ In addition to the lack of sustained comprehensive malaria control activities, there was severe flooding in 2012 in riverine communities in Loreto, which led to an increase in the number of malaria cases. In 2017, the Peruvian government established a long-term initiative called the Malaria Zero Plan that was initially focused on Loreto, where a reduction of malaria cases by 70% was achieved in 2020.^4^ Finally, in 2022, the plan became national, with the aim of reducing malaria cases by 90% by 2030.^5^

Previous studies by the Amazonian International Center of Excellence for Malaria Research (Amazonian-ICEMR) highlight the microheterogeneity of malaria transmission in two different environmental settings less than 2 hours from Iquitos, the capital of Loreto Department: Lupuna, a riverine community located on the outskirts of Iquitos, and Cahuide, located on both sides of the Iquitos-Nauta road.^6^^,^^7^ Some studies in rural riverine communities found heterogeneity in malaria transmission, which was interpreted to arise from interactions among the microgeographic landscapes driving diverse conditions for vector development; housing structure and location; and other sociodemographic factors, like age, education, travel, and occupational activities.^8^^,^^9^ However, no studies have been conducted in remote, hard-to-reach communities to examine malaria transmission dynamics, particularly from a parasite population genetics perspective. Here, we conducted a retrospective analysis to calculate the prevalence of malaria infections by quantitative real-time polymerase chain reaction (qPCR) and to measure genetic diversity and population structure of *Plasmodium vivax* in Santa Emilia, a remote, hard-to-reach riverine community in the Peruvian Amazon region, in 2015–2016. To test the hypothesis that genetic diversity of *P. vivax* was maintained in different short-term study periods, we compared these results with genetic data from 2013. A bottleneck and relatedness analysis between parasites allowed us to gain insights into the dynamics of malaria transmission in this remote community.

## MATERIALS AND METHODS

### Study site and *P. vivax* sample collection.

This study was conducted in the Santa Emilia community, which is located in Loreto Department of the Peruvian Amazon region (Supplemental Figure 1). Santa Emilia (04°11′58.99″S, 74°12′20.12″W) is a remote site accessible only by small boats by the Nahuapa Stream, and it is approximately 150 km from Iquitos, the capital of Loreto. The community has a population of 213 individuals distributed across 50 houses. It lacks electricity and basic sanitation services, such as water and sewage, and it also does not have a health center. The population primarily engages in fishing, hunting, and agriculture as subsistence activities (data observed during the census).

The Amazonian-ICEMR conducted a project in Santa Emilia (“Malaria transmission in remote communities in the Amazon region of Peru—phase II”) from May 2015 to May 2016, which included monthly surveillance activities.

Finger-prick blood samples were collected on slides and filter paper for malaria diagnosis by microscopy and qPCR, respectively. Weekly active case detection and monthly population screening were the methods to collect the samples, independent of whether there were symptoms during the monthly surveillance. Positive malaria cases detected in situ by microscopy were immediately referred to receive treatment according to the national guidelines from the Peruvian Ministry of Health (MoH).^10^ Samples collected on filter paper were processed later in our laboratory in Lima, Peru for malaria molecular diagnosis and genotyping.

Data from 139 *P. vivax* samples collected and genotyped using 16 neutral and unlinked microsatellites in a previous study in Santa Emilia^11^ were used to compare the parasite population changes of diversity and population structure with samples from 2015 and 2016 used in this study. The same 16 microsatellites, described previously,^11^ were used in this study.

### DNA extraction, qPCR diagnosis, and microsatellite genotyping.

During 2015 and 2016, 3,434 blood samples were collected on filter paper for molecular diagnosis and genotyping. *Plasmodium* DNA was isolated using the DNA Mini Kit and E.Z.N.A. Blood DNA kit (OMEGA Bio-tek, Norcross, GA) following the manufacturer’s instructions. Genomic DNA was eluted in 50 *µ*L of elution buffer and stored at −20°C until its use in subsequent molecular analyses. Molecular diagnosis by qPCR was performed following the protocol by Mangold et al.^12^ that was modified by Manrique et al.^11^

The absolute quantification of parasite DNA concentration was done using the protocol published by Manrique et al.^11^ Based on this protocol, 552 *P. vivax*-positive samples were categorized according to their parasitemia. A total of 107 samples with parasitemia >24 molecules/*µ*L were diluted to 8 molecules/*µ*L; 66 samples were within the range of 4–24 molecules/*µ*L and were reamplified using the Illustra GenomiPhi V2 DNA amplification kit (New England Biolabs, Ipswich, MA) and the protocol proposed by Cowell et al.^13^ Seventy-seven samples were into the range of 1.5–3 molecules/*µ*L, and we had to repeat the DNA extraction; however, only 67 were genotyped. A total of 312 samples had parasitemia below 1.5 genome equivalents/*µ*L and were excluded from the analysis (Supplemental Figure 2).

In total, 240 samples were genotyped with the same 16 microsatellites previously published by Manrique et al.,^11^ Karunaweera et al.,^14^ and Imwong et al.^15^ (Supplemental Table 1), and to evaluate the presence of alleles and null alleles, 2% agarose gels were used. The discrimination of alleles in the samples was done by capillary electrophoresis in the ABI PRISM 3100 system from Applied Biosystems (Foster City, CA) using the GeneScan 500 LIZ base-pair marker and the peak scanner v. 10 program (Applied Biosystems). The principal alleles and any additional alleles with peak height at least one third of the height of the predominant alleles per locus were scored. Only 221 samples amplified at least 10 microsatellites; this number of microsatellites was enough to discriminate nearly 100% of all haplotypes found. Additionally, 139 samples collected in 2013 and 221 samples from 2015 and 2016 were used to perform further analyses (Supplemental Figure 2).

## STATISTICAL ANALYSES

All statistical analyses were conducted in R v. 4.0.4 (R Development Core Team, R Foundation for Statistical Computing, Vienna, Austria). The metrics of diversity genetics, population differentiation, multiplicity of infection (MOI), polyclonality, and population structure were analyzed by year (2013, 2015, and 2016) to evaluate changes over time.

To determine whether there were statistically significant changes in genetic diversity (Index Nei) obtained for each sampling year, a Monte Carlo test was performed with 1,000 resamplings and a statistical significance threshold (*P* <0.05). Additionally, the population mutation rate (θ), the standardized index of association, the number of multilocus genotypes, the number of effective alleles, and MOI were compared over time with the Kruskal–Wallis test. Finally, the proportion of polyclonal infections was compared using the Fisher exact test. Samples of 2015 and 2016 were pooled into a single group to achieve a similar sample size to 2013; these were used for bottleneck and minimum expanding network analyses.

### Metrics of genetic diversity.

Genetic diversity was measured using Index Nei (He=1-∑pi2), and the effective numbers of alleles ($N_{e}^{a}$) were calculated by inverting the measure of homozygosity in a locus in each population ($1/\sum_{pi}2$). Linkage disequilibrium (LD) was measured as indirect inbreeding in the population. The standardized index of association was used as a summary measure of LD. We used single-clone haplotypes to avoid false inbreeding because of clonal propagation; the significance of the *I*_A_^S^ estimates was assessed using 10,000 random permutations of the data. These analyses were performed using poppr packages in R. The population mutation rate (*Theta_h_* = θ) was performed using the Pegas package implemented in R. A Monte Carlo test was performed to confirm whether significant differences between samples collected in different years exist.

### MOI and polyclonality.

Multiplicity of infection was defined as the presence of one or two alleles in the same genetic locus. The MOI average was calculated as the ratio of the total number of distinct fragments (genetically distinct parasite clones) scored for a given locus in relation to the number successfully amplified by that locus marker.^16^ It is important to emphasize that this calculation was performed on a per-sample basis, ensuring that the number of fragments considered reflects the genetic diversity within each individual sample rather than an aggregation across all samples. The polyclonality was defined if at least two alleles were different in the same infection.

### Bottleneck analysis.

To evaluate a recent demographic changes (bottleneck effect) in the *P. vivax* population, the Bottleneck Program v. 1.2.02 was used.^17^ The Wilcoxon test was used to evaluate heterozygosity excess or deficit, and the expected heterozygosity in an equilibrium population was estimated using 10,000 iterations. This analysis was conducted with 13 perfect microsatellites and two different mutational models; the stepwise mutation model (SMM) and the two-phase model (TPM) were performed. In total, 139 samples analyzed previously (2013) and 221 collected during 2015–2016 were analyzed with this test.

### Population differentiation and population structure.

The degree of population differentiation was measured by calculating the Jost *D*.^18^ The comparison was made among surveillance years, specifically 2013, 2015, and 2016. The mmod package^19^ implemented in R was used to determine the Jost index. Additionally, an analysis of molecular variance (AMOVA) was conducted to evaluate the sources of population differentiation using the ade4 R package.^20^ The statistical significance of fixation indexes (phi) was assessed using 10,000 permutations.

Structure v. 2.3 was used to determine the number of populations present in the study area. An admixture model was applied with 10 replicates for each value of *k* (ranging from 1 to 10) and a burn-in period of 50,000 followed by 200,000 iterations of Monte Carlo Markov chains to obtain the optimal number of populations. The second-order rate of change in marginal likelihood (ΔΔ*K*), as described by Evanno et al.,^21^ was used in the Structure Harvester program to estimate the number of populations present. Graphical visualization of the populations and ancestry coefficients was performed using the CLUMPLAK program.^22^

### Minimum expanding network of *P. vivax*.

The genetic relatedness between haplotypes was measured in a function of genetic distance. The distant of Bruvo et al.^23^ was used in poppr implemented in the R program. The distance between two individuals of Bruvo et al.^23^ calculates the minimum distance across all combinations of possible pairs of alleles at a single locus and then, averages that distance across all loci. In this work, two haplotypes are related if they have a Bruvo genetic distance less than 0.5. The minimum expanding network was inferred assuming the parsimony principle.

## RESULTS

### Prevalence of *P. vivax* and submicroscopic infections during 2015–2016.

**Figure 1.**
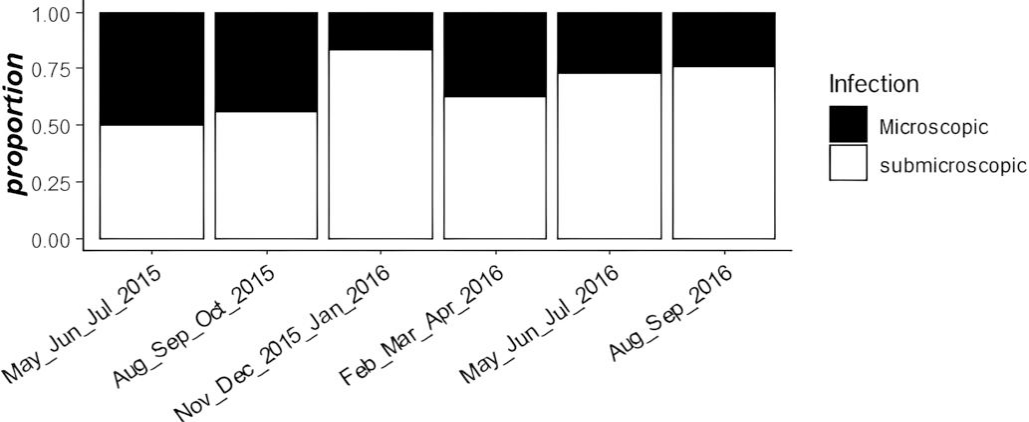
Proportions of submicroscopic/microscopic infections during 2015 and 2016. The *x* axis represents the time grouped by 3 months between 2015 and 2016. The *y* axis represents the proportion of submicroscopic/microscopic infections.

The prevalence of *P. vivax* by qPCR was 56%, almost 2-fold higher than that determined by microscopy (30%). The Fisher exact test showed a statistically significant difference (*P* <0.05) between these periods. Additionally, we measured the proportion of submicroscopic and microscopic infections during 2015 and 2016, and these results showed an increase between 50% and 75% of submicroscopic infections over time (Figure 1). In general, the average prevalence of *P. vivax* submicroscopic infections was 44%.

### Genetic diversity metrics: A comparison between the 2013 and 2015–2016 surveillance periods.

Genetic diversity average (*H*_exp_ ± [standard error] = 0.707 ± 0.109) was high in both surveillance periods (2013 and 2015–2016) (Table 1), and it did not show statistically significant differences (*P* >0.05). In the same way, the mean MOI was 1.066, and LD (*I*_A_^S^ = 0.118) was similar in both surveillance periods, with no statistically significant difference (*P* >0.05) using the Kruskal–Wallis test.

**Table 1:** Genetic diversity metrics from the surveillance

Year	*N*	*H*_exp_ (SE)	*Theta_h_* (SE)	*I*_A_^S^	MLG	$N_{e}^{a}$	MOI	Polyclonal (95% CI)
2013	139	0.595 ± 0.147	1.919 ± 0.438	0.181	113	2.470	1.074	0.41 (0.33–0.49)
2015	150	0.619 ± 0.192	1.623 ± 0.578	0.129	145	2.626	1.076	0.46 (0.33–0.54)
2016	71	0.636 ± 0.187	1.765 ± 0.711	0.106	69	2.750	1.048	0.29 (0.20–0.41)
Total	360	0.707 ± 0.109	4.284 ± 1.075	0.118	319	3.417	1.066	0.39 (0.34–0.47)

*H*_exp_ = expected heterozygosity; *I*_A_^S^ = standardized index of association (all *P*-values were below 0.002 [Ho (null hypothesis): *I*_A_^S^ = 0]); MLG = number of multilocus genotypes; MOI = multiplicity of infection; *N* = sample size; $N_{e}^{a}$ = effective numbers of alleles; polyclonal = proportion of polyclonal infections; SE = standard error; *Theta_h_* = population mutation rate based on the expected homozygosity.

In total, 113 haplotypes were found 2013, 145 haplotypes were found in 2015, and 69 haplotypes were found in 2016. During 2013, the proportion of polyclonal infections was 41%. In 2015, it increased to 46%; however, in 2016, this proportion decreased to 29%. The Fisher exact test did not show statistically significant differences between the proportions of polyclonal infections in 2013 compared with 2015–2016 (*P* >0.05). Additionally, the number of effective number of alleles ($N_{e}^{a}$) and the population mutation rate (*Theta*) did not change during both surveillance periods, and they did not show statistically significant differences (*P* >0.05) (Table 1).

### Population differentiation and population structure during 2013 and 2015–2016.

We found high pair-wise genetic differentiation among the years 2013 and 2015–2016 (Jost *D* >0.5) but low genetic differentiation from 2015 to 2016 (Jost *D* = 0.009) (Supplemental Figure 3). These outcomes were confirmed by the AMOVA, where just 13.0% of the genetic variation was explained between years, and 87.1% was explained by differences within years of surveillance (Supplemental Table 2). These results suggest the presence of unique haplotypes that appeared in each year of surveillance. We detected *P. vivax* population structuring during all study periods. The number of subpopulations was estimated to be four (Supplemental Figure 4). During 2013 surveillance, four clonal populations were probably present (Δ*K* = 4), and in 2015–2016, four different clonal populations were also present. Additionally, more admixture was showed during 2015–2016 (Supplemental Figure 5). These findings support the hypothesis that the Santa Emilia community is receptive to different clonal populations.

### Bottleneck analysis based on two different mutations models.

The bottleneck analysis using 13 polymorphic markers showed a significant number of microsatellites with an heterozygosity (He) deficiency during 2013, 2015, and 2016 under SMM (Wilcoxon test one side and two sides: *P* <0.05) and TPM (Wilcoxon test one side and two sides: *P* <0.05) models, indicating that an event heterozygosity deficit may result from a recent expansion of the population size. Twelve loci with heterozygosity deficiency and 1 locus with heterozygosity excess were found under the SMM model, and 11 loci with heterozygosity deficiency and 2 loci with heterozygosity excess were found under the TPM model (Table 2). The mode shift analysis showed that the allele frequencies of the populations did not exhibit a change in the L-shaped distribution, suggesting a mutation drift equilibrium and the absence of a recent bottleneck (Supplemental Figure 6).

**Table 2:** Bottleneck test before and during the surveillance according two mutations models

Surveillance	Population	Size of Population	Mean Number Alleles by Locus	Mutation Model	Loci Deficit	Loci in Excess	Event	Test of 1 Side (*P*-Value)		Test of 2 Sides (*P*-Value)	
								SMM	TPM	SMM	TPM
Before	2013	134.15	6	SMM	7	6	Excess heterozygous	0.99957	0.99738	0.00122	0.00671
				TPM	11	2	Deficit heterozygous	0.00061	0.00336		
During	2015	137.92	10.15	SMM	13	0	Excess heterozygous	1	1	0.00012	0.00012
				TPM	13	0	Deficit heterozygous	0.00006	0.00006		
	2016	65.62	8.08	SMM	11	1	Excess heterozygous	0.99994	0.99988	0.00024	0.00037
				TPM	12	1	Deficit heterozygous	0.00018	0.00018		

Deficit heterozygous = significant genetic diversity deficiency; excess heterozygous = significant genetic diversity excess; SMM = stepwise mutation model; TPM = two-phase model.

### Minimum expanding network of *P. vivax*.

During the 2013 and 2015–2016 surveillance, we found closely genetically related parasites. This result suggests a clonal expansion of *P. vivax* during all surveillance. Also, a few haplotypes from 2013 were found during 2015; in the same way, several haplotypes that were genetically related were present in 2015–2016 (Supplemental Figure 7). These findings provide evidence of continuous local transmission in Santa Emilia, with some haplotypes circulating throughout the entire surveillance period.

## DISCUSSION

We found a high proportion of microscopic and submicroscopic *P. vivax* infections in the remote community of Santa Emilia in 2015–2016, with high genetic diversity (*H*_exp_ = 0.63 ± 0.18 and *H*_exp_ = 0.59 ± 0.14, respectively) and high population differentiation (Jost *D* >0.5) compared with 2013. We also observed a year-by-year, short-term change in parasite populations that is consistent with previous studies from small geographic areas in Thailand, central China, Colombia, Brazil, and Venezuela,^24^^–^^28^ where high genetic diversity and substantial population differentiation^11^^,^^29^^,^^30^ have been found. This seems characteristic of a heterogeneous population structure resulting from complex regional dynamics.^8^

The high proportion of submicroscopic infections found is likely because of the low parasitemia and asymptomatic relapses from hypnozoites related to the biological behavior shown by *P. vivax*. This characteristic can be explained by its preference for invading younger reticulocytes to develop schizont stages.^31^ Several studies revealed a high burden of submicroscopic infections in areas with variable *P. vivax* epidemiological scenarios.^32^^–^^34^

Furthermore, studies in different communities from the Peruvian Amazon have shown 80% submicroscopic infections,^8^^,^^34^ including in communities from Mazan and Lupuna.^6^^,^^35^ These communities are characterized by persistent residual and microheterogeneous transmission, which is facilitated by their connectivity by river and road with other communities.

Submicroscopic infections are challenging for standard control strategies based on passive case detection using microscopy, which continue to be the practice in Peru. These undetected infections likely act as a silent reservoir that contributes to maintaining transmission.^32^^–^^34^ New and more sensitive diagnostic tools should be implemented to detect these infections effectively. For example, loop-mediated isothermal amplification has been suggested as a potentially valuable tool in Peru because it is particularly easy to implement in rural and remote areas with high prevalence of submicroscopic asymptomatic infections.^36^^,^^37^

It is worth noting that despite Santa Emilia’s remote, hard-to-reach location, we cannot rule out the potential influx of parasites into the community because of internal regional travel given the high mobility observed within it, particularly related to occupation (survey data are currently in preparation for publication). This hypothesis is supported by prior studies, which demonstrated that Santa Emilia maintained genetic connectivity with parasites from geographically distant transmission sites, such as Lupuna and Cahuide communities.^11^ It is also likely that subclinical malaria infections are being imported from closer communities, such as 28 de Julio, El Cerro, and Victor Raul. Still, more studies are needed to demonstrate this hypothesis. In Santa Emilia, commercial trips to Nauta are common; individuals who extract wood go to the water source outside their community, and household overcrowding, with more than five people sleeping in a house (observed in a recent survey), could contribute to maintaining the transmission. Such travelers should be considered a target of malaria control.^38^^–^^41^

The presence of high proportions of polyclonal infections (>40%) in 2013 and 2015 is comparable with other studies reported in the Peruvian Amazon and areas with overall high transmission.^11^^,^^28^^,^^30^ However, these proportions contrast with findings from other continents.^25^^,^^42^^,^^43^ Polyclonal infections can be explained by the dynamics of relapses, which were not evaluated in this study. The reduction in the proportion of polyclonal infections in 2016 can be attributed to the sample size included in this study (*n* = 71) because of the high number of submicroscopic infections with very low parasitemia that were not included in the genotyping analysis. This is an important limitation of the study. Although interpreting MOI is complex,^28^ we found that it ranged between 1.07 and 1.04, comparable with a study conducted in other rural areas of the Peruvian Amazon but low compared with other countries.^25^^,^^42^

Based on the preliminary results of the present work, the Peruvian MoH implemented standard control interventions in this community, including indoor residual spraying (March 2012, March 2013, and October 2014), bed net distribution (Supplemental Table 3), active case detection, and microscopy-based treatment.^44^ Despite these efforts, malaria prevalence remained unchanged as observed in this study. This finding aligns with similar findings from two other communities in the Peruvian Amazon with contrasting epidemiological scenarios.^6^ The intermittent nature of these interventions in Santa Emilia may have facilitated the reintroduction of *P. vivax* infections in 2015–2016, potentially explaining the observed parasite replacement over time. These findings underscore the necessity of designing comprehensive and sustained malaria control.^45^

A decrease in parasite genetic diversity or other population genetic metrics would be expected concomitant with the sustained reduction of the parasite population in response to interventions performed by the Peruvian MoH.^45^ However, *P. vivax* seems resilient to these interventions. Other studies reporting high genetic diversity and complex population structure in scenarios with low transmission intensity have reported similar patterns to the one reported here.^27^^,^^30^^,^^41^^,^^46^

Currently, the Peruvian Amazon exhibits a different epidemiological scenario. Microscopic malaria cases reported by the Peruvian MoH have declined by approximately 75% compared with 2018. This significant reduction of microscopic cases is also evident in Santa Emilia (Supplemental Table 4); however, standard control measures are insufficient to detect submicroscopic infections and hypnozoite reservoirs, which pose a risk for malaria resurgence. In this context, the malaria elimination plan should incorporate continuous, long-term interventions using novel tools to identify submicroscopic infections and recent exposures, particularly targeting mobile populations acting as parasite carriers.^45^

## CONCLUSION

In conclusion, during 2015 and 2016, high proportions of *P. vivax* submicroscopic infections were detected accompanied by significant genetic diversity compared with infections from 2013. Additionally, there was very high population differentiation in Santa Emilia, a remote community in the Peruvian Amazon region. Absence of a recent bottleneck and rapid clonal expansion were also observed. Despite geographical isolation, our results indicate that *P. vivax* malaria in Santa Emilia maintains high local transmission and persists despite standard interventions, keeping the community at elevated malaria risk. Thus, eliminating *P. vivax* poses different challenges than eliminating *Plasmodium falciparum*, mainly because of its biological characteristics, including relapse owing to liver stages, the predominance of low and asymptomatic parasitemia, and higher genetic diversity.^47^^,^^48^ Implementing frequent and sustainable interventions in communities with limited access is a priority for malaria elimination.

## Supplemental Materials

10.4269/ajtmh.24-0662 Supplemental Materials
