# Supplementary material for: High Prevalence, Genetic Diversity and Temporal Differentiation of Plasmodium vivax in a Remote Hard-to-Reach Community from the Peruvian Amazon Region
Source: Am J Trop Med Hyg. 2025 Aug 19;113(5):990–6. doi: 10.4269/ajtmh.24-0662 (PMC12590998; doi:10.4269/ajtmh.24-0662)
Supplement: Supplemental Materials [file tpmd240662.SD1.pdf]

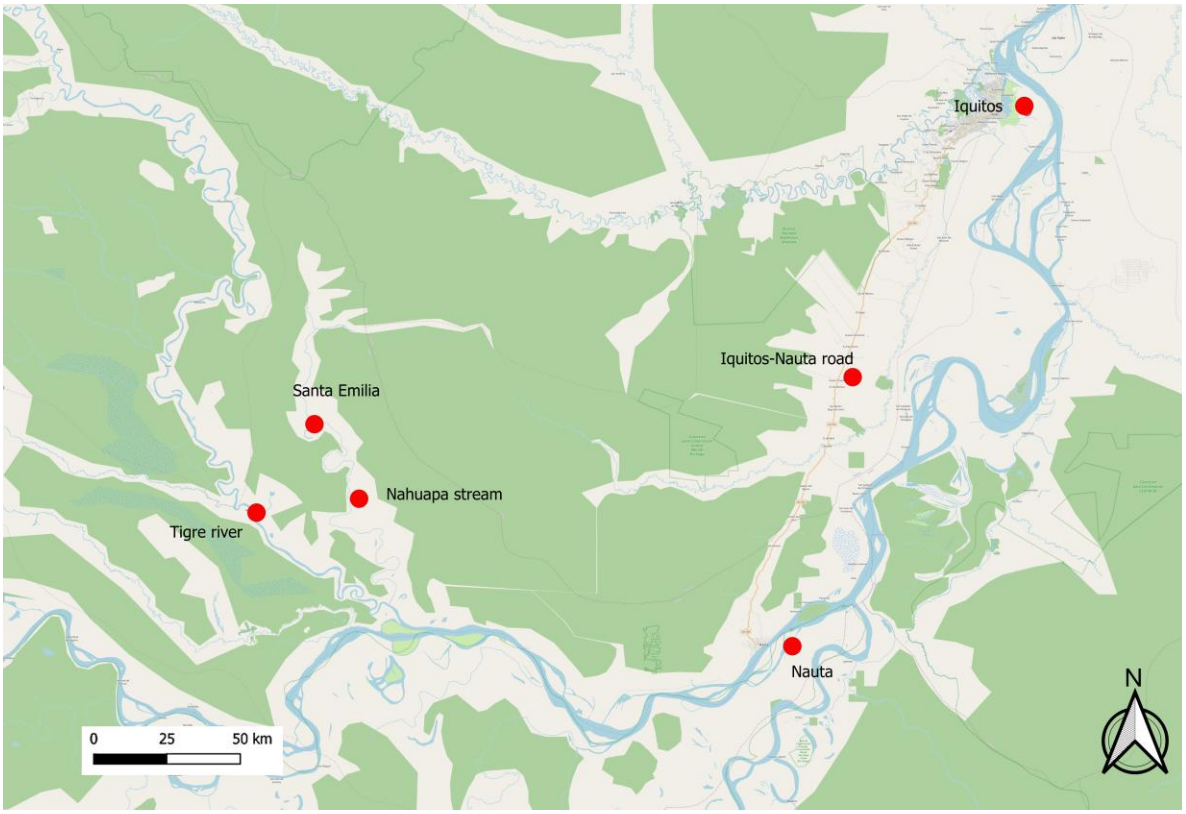

Figure S1. Geographic location of the Santa Emilia community in Loreto department. This map was produced using QGIS development Team 2.18.14 (2018).

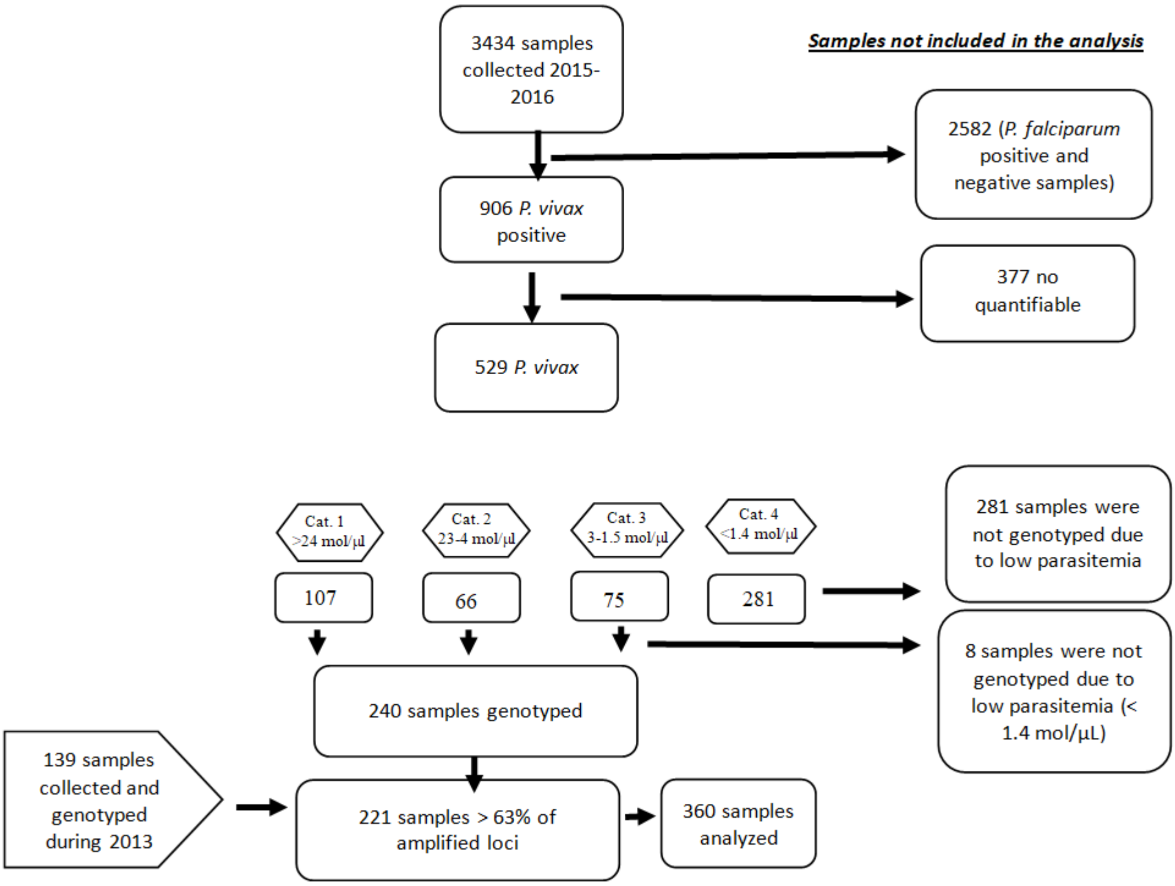

Figure S2. Sample selection and processing flowchart: During 2015–2016, a total of 3,434 samples were collected. Of these, 906 *P. vivax*-positive samples were used for prevalence analysis. Only 529 of them were quantifiable (molecules/µL) and were classified into four categories (Cat 1, 2, 3, and 4). A total of 240 samples were genotyped, but only 221 amplified more than 61% of the loci; only these samples were used for genetic analysis, along with 139 samples genotyped in 2013.

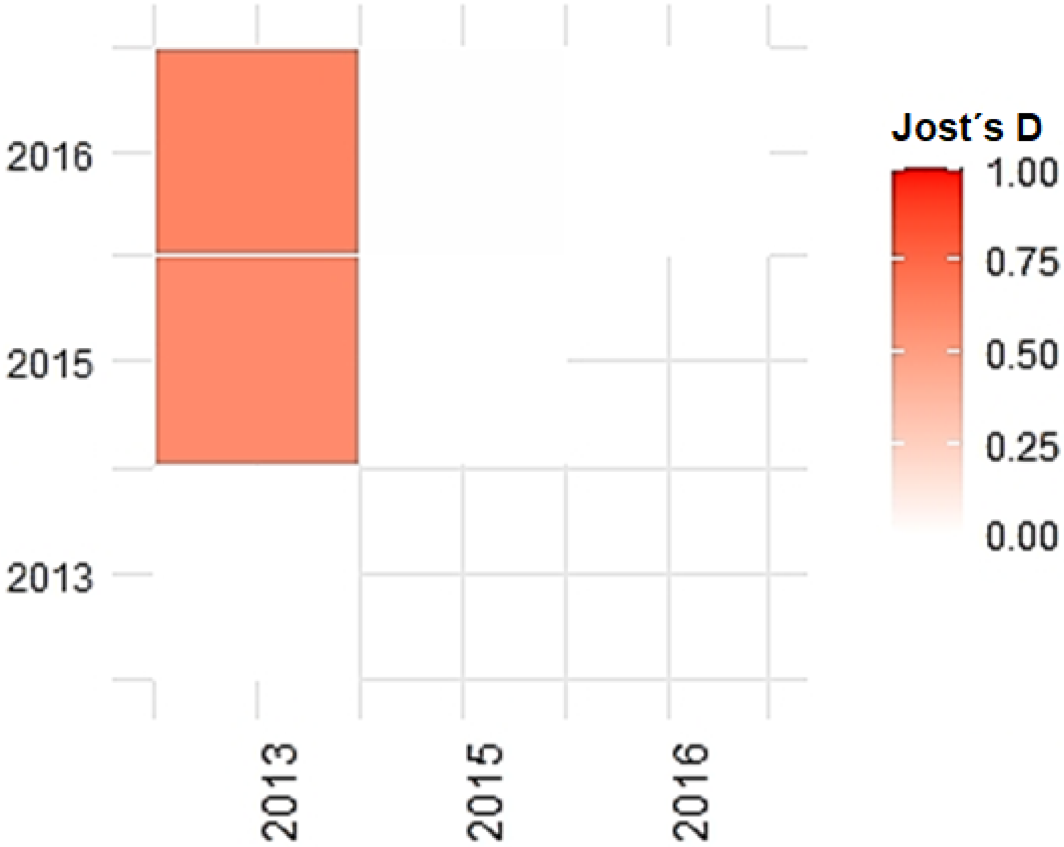

Figure S3. Population Differentiation between pairwise years collected of *P. vivax*. The pairwise population differentiation proposed by Jost L. (2008). The increase in color scale represents greater population differentiation.

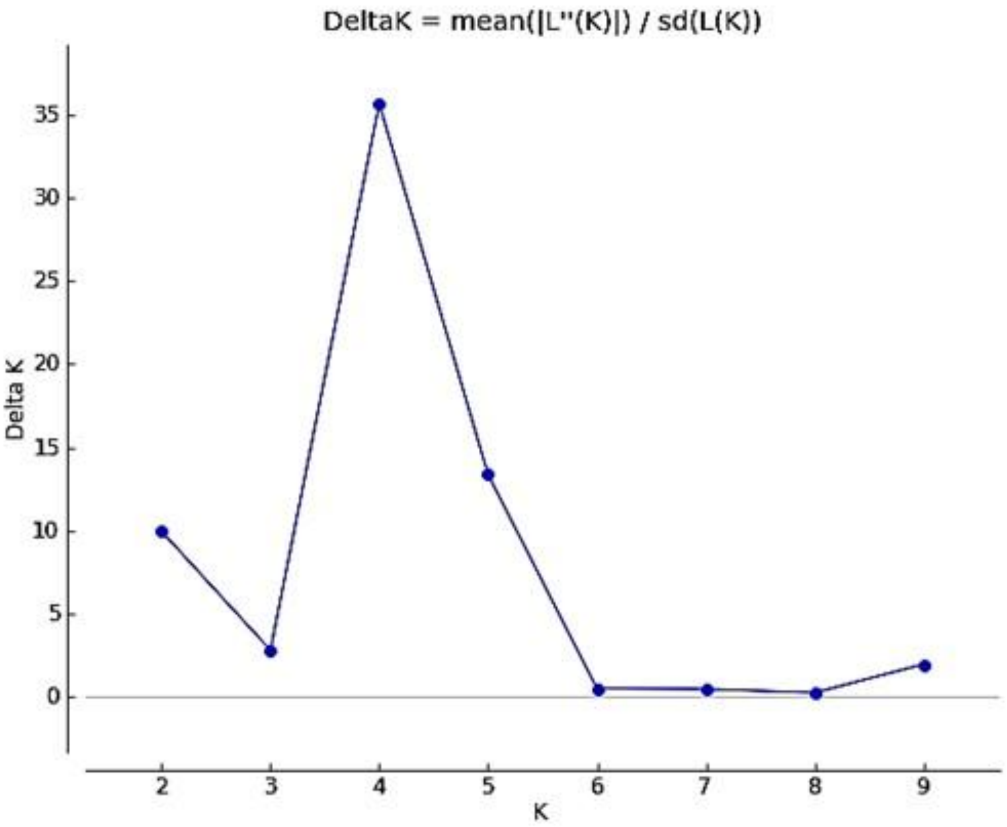

Figure S4. Analysis of the second order rate of change in the logarithmic marginal likelihood proposed by Evanno, Regnaut. The higher value of k denotes the number more likely populations.

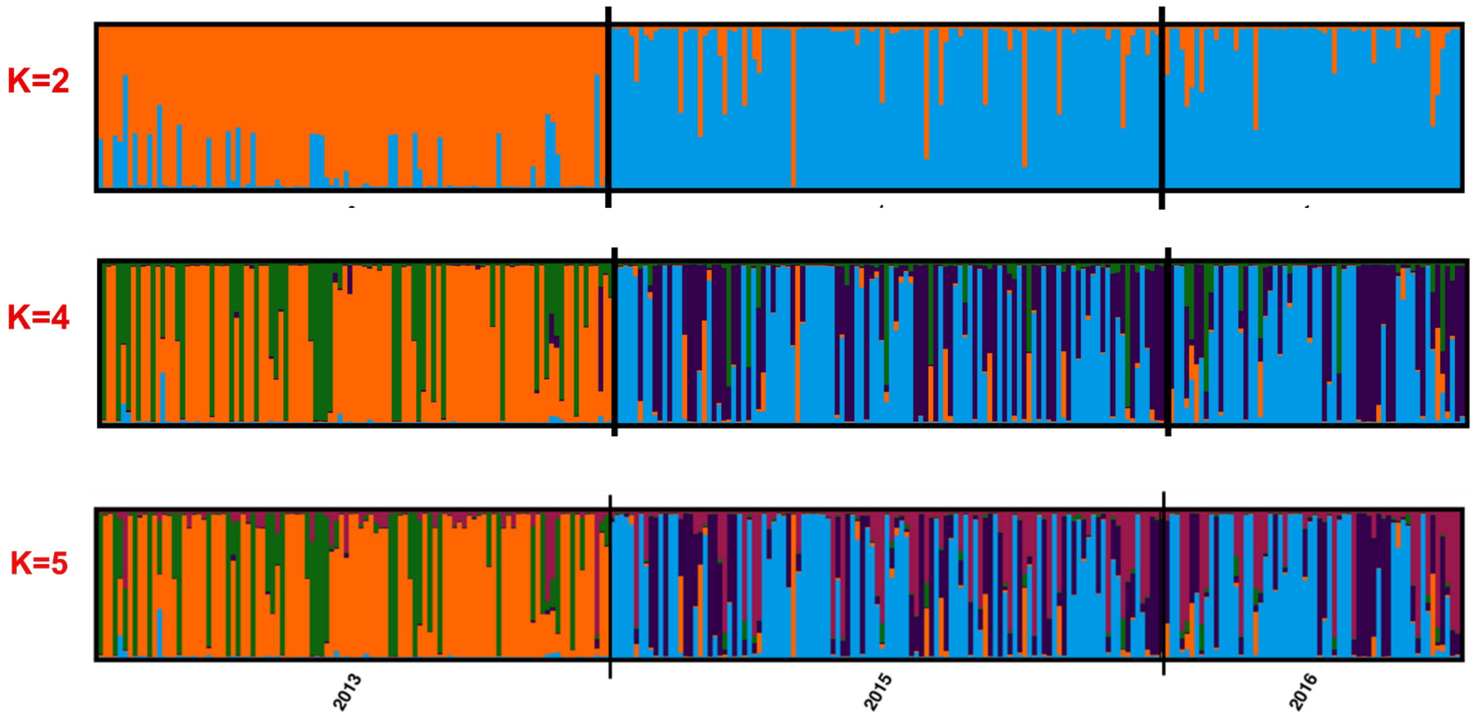

Figure S5. Population structure of *P. vivax* inferred using the STRUCTURE software

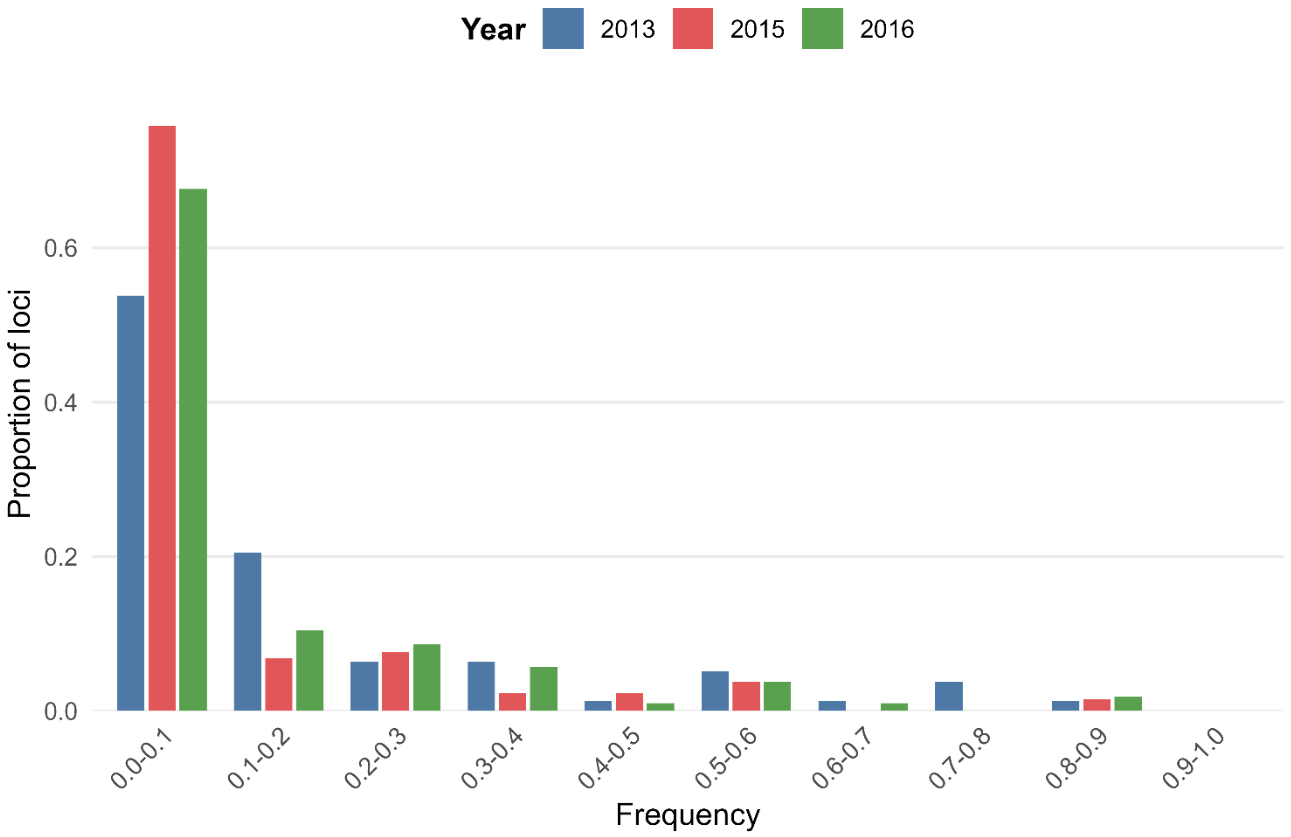

Figure S6. Mode-shift analysis of allele frequency distribution.

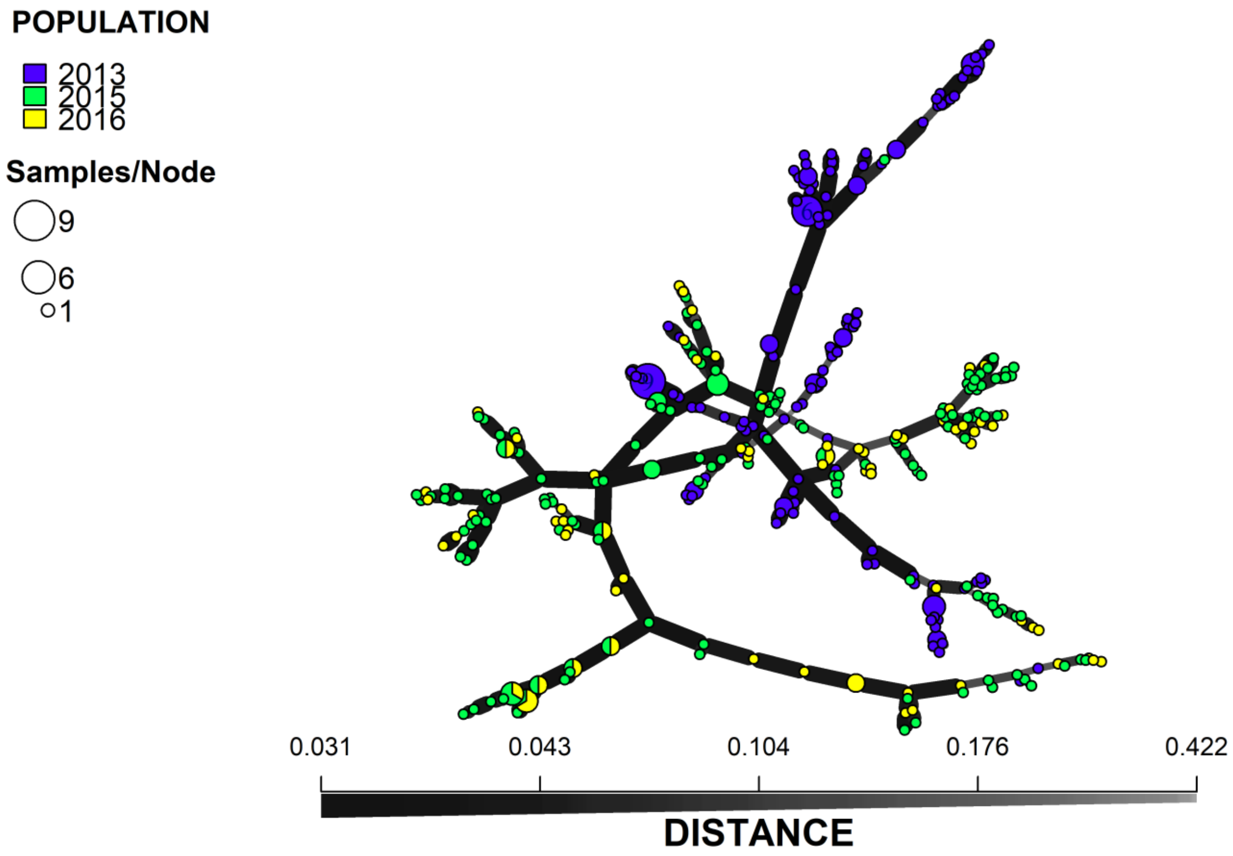

Figure S7. Minimum expanding network of *P. vivax.*

Table S1
Information on sixteen microsatellites used

MS	Chromosome	start	Tandem repeat	Size (pb)	Primer Forward	Primer Reverse
11.162	Pv_Sal_chr11	1859261	ATAC	222	6FAM-TTTGTTAGGAGATCCGTCTG	TAAATGACACTTTGGCTTCC
14297	Pv_Sal_chr14	2965458	AAG	200	PET-TACACCCTTTAGGTCCTCGT	TGAAAAATGTTCCGCTACTT
Ch2.121	Pv_Sal_chr02	121392	TTA	172	NED-CCTTGCTCTCCTGGCTCTTC	GTATGAGTGACCACCCTGGC
Ch2.152	Pv_Sal_chr02	152435	AAT	153	VIC-GTCATTTTCTAGGCAAAGACAGC	TACGTTTCGCCATGTTCCTG
Ch14.2986	Pv_Sal_chr14	2986814	AT	175	NED-TTCCTAAATAAAGCACCATG	ACCATTCTGTTAAAATGTATGT
Ch14.3010	Pv_Sal_chr14	3010226	AT	207	PET-GATAAGATAAAACAACAAATGCG	AGGATGCTTCGGTTTGCT
Ch14.3021	Pv_Sal_chr14	3021021	TA	228	6FAM-GCATTTGTTACATTGTTTTCGT	GCAAGATGTGTCCTTTACA
Ch2.122	Pv_Sal_chr02	122021	ATA	197	VIC-AGCTGGATTGCCCTAACTGC	CGCATCATCGCATACGCTTG
13.239	Pv_Sal_chr13	1601741	TTTA	210	NED-CAACAACAAAATGAATGACG	CCTACCTTTACGCGTTTTTA
Ch14.2981	Pv_Sal_chr14	2981291	AT	161	PET-CGATGAATCATTTTCGTCCGCA	ACTCTTCTTTCAGCACTTTTTGCC
MS6	Pv_Sal_chr11	1760698	(TCC)2(TCT)3(CCT)2(TCC)2GCTTCT(TCC)10	241	VIC-GGTTCTTCGGTGATCTCTGC	CTGTCTTGGAGGACATCAACGGGATT
MS9	Pv_Sal_chr08	1394242	(GGA)18	188	6FAM-AGATGCCTACACGTTGACGA	CTGTCTTGAAGCTGCCCATGTGGTAAT
3.502	Pv_Sal_chr03	451099	AACGGATG	168	PET-GTGGACCGATGGACCTAT	TCCTACTCAGGGGGAATACT
MS15	Pv_Sal_chr05	1110660	(TCT)10	243	NED-TGTTTGCAAAGGAATCCACA	CTGTCTTCGGCCAGATGAAAAGGATAA
MS20	Pv_Sal_chr10	1345613	(GAA)11GAG(GAA)13(CAA)4GAA(CAA)5	211	VIC-GCACAACAAATGCAAGATCC	CTGTCTTGTGGCAGTGGCTCATCTTCT
MS4	Pv_Sal_chr06	390261	(AGT)18	220	6FAM-CGATTTACTGTTGACGCTGAA	CTGTCTTCAAAGGAACATGCTCGATGA

Table S2
Analysis of molecular variance (AMOVA) by sampling year

Source of variation	df	SS	MS	Sigma	Variation (%)	phi (φ)	p-value
Between years	2	9.087	4.544	0.037	12.95	**0.016**	**0.002**
Within years	357	89.605	0.251	0.251	87.05	0.129	**0.001**
Total	359	98.691	0.275	0.288	100		

df: degree of freedom; SS: sum of square; MS: mean sum of square

Table S3
Intervention performed by the MoH since 2012 until 2019 in Santa Emilia community

**Year**	**2012**	**2013**	**2014**	**2017**	**2019**
Spraying indoor	March	March	October	May	Not performed
Bed net distribution (92% coverage rate)	Not performed	Not performed	Not performed	Not performed	October

TABLE S4
Microscopic cases reported by the MoH in Peru from 2013 to 2022 in the Santa Emilia community

Año	*P. falciparum*	*P. vivax*	Total
2013	46	132	178
2014	26	178	204
2015	76	176	252
2016	4	33	37
2017	9	48	57
2018	1	23	24
2019	0	9	9
2020	0	6	6
2022	0	2	2
